# Review of the novel antifungal drug olorofim (F901318)

**DOI:** 10.1186/s12879-024-10143-3

**Published:** 2024-11-07

**Authors:** Yuri Vanbiervliet, Tine Van Nieuwenhuyse, Robina Aerts, Katrien Lagrou, Isabel Spriet, Johan Maertens

**Affiliations:** 1 Department of Haematology, Department of Microbiology, Immunology and Transplantation, University Hospitals Leuven, KU Leuven, Herestraat 49, Leuven, 3000 Belgium; 2 Pharmacy Department, University Hospitals Leuven, Herestraat 49, Leuven, 3000 Belgium; 3 Department of Laboratory Medicine and National Reference Center for Mycosis, Department of Microbiology, Immunology and Transplantation, University Hospitals Leuven, KU Leuven, Herestraat 49, Leuven, 3000 Belgium; 4 Department Of Pharmaceutical and Pharmacological Sciences, Pharmacy Department University Hospitals Leuven, KU Leuven, Herestraat 49, Leuven, 3000 Belgium

**Keywords:** Novel antifungal therapy, Dihydroorotate dehydrogenase, Olorofim, F901318, Invasive fungal diseases, Aspergillus, Lomentospora, Scedosporium, Coccidioides

## Abstract

There is clearly a need for novel antifungal agents, not only concerning spectrum, but also oral bioavailability, tolerability, and drug-drug interactions. There is growing concern for antifungal resistance for current available antifungals, mainly driven by environmental fungicide use or long-term exposure to antifungals, in the setting of mould-active prophylaxis or for chronic antifungal infections, such as chronic pulmonary aspergillosis. Moreover, the incidence of breakthrough infections is increasing, because of the introduction of (mould-active) prophylaxis (1-4). There is emergence of difficult to treat invasive fungal infections, such as those caused by *Lomentospora prolificans*, cryptic species of *Aspergillus*, *Scedosporium* and *Coccidioides*. Olorofim (F901318) is the first-in class of the orotomides, a novel antifungal class targeting dihydroorotate dehydrogenase (DHODH), a key enzyme in the biosynthesis of pyrimidines. Olorofim shows good in vitro and in vivo activity against *Aspergillus* species, rare and difficult to treat moulds and endemic dimorphic fungi, including azole- and amphotericin-resistant isolates. It lacks activity against yeasts and the *Mucorales* species. It is only orally available and shows very promising results in ongoing clinical trials. In this review we will describe the mechanism of action of olorofim, the spectrum of activity in vitro and in vivo, pharmacokinetics, pharmacodynamics, drug-drug interactions, resistance, and clinical outcomes.

**Supplementary Information:**

The online version contains supplementary material available at 10.1186/s12879-024-10143-3.

## Background

In 2022, the WHO published their first fungal priority pathogen list. *Candida albicans*,* Aspergillus fumigatus*,* Candida auris*,* Cryptococcus neoformans* are deemed of critical importance. Fusarium spp.,* Candida tropicalis*,* Candida parapsilosis*,* Candida glabrata*,* Histoplasma capsulatum* and the fungi causing mucormycosis or mycetoma are deemed of high importance [5]. Aspergillus species are the most important pathogen of invasive mould infections, affecting more than 2.100.000 people annually worldwide [6–8]. There is emergence of difficult to treat infections such as those caused by *Lomentospora prolificans*, cryptic species of *Aspergillus*, *Mucorales* and other rare invasive fungal diseases (IFDs) [5, 9–22]. Survival of patients with invasive aspergillosis (IA) has improved over the last two decades since the introduction of triazoles as first-line therapy and because of improved diagnostics and supportive care [23–27]. Patients with severe neutropenia and severe graft-versus host disease (GVHD) are at particular high risk. However, IFDs are emerging with more immunocompromised and non-immunocompromised patients at risk, partly due to novel anti-cancer therapies and more solid organ transplantation but also in critical ill patients with severe (viral) pneumonia and in patients with chronic obstructive pulmonary disease (COPD) [8, 28–36].

**Fig. 1:**
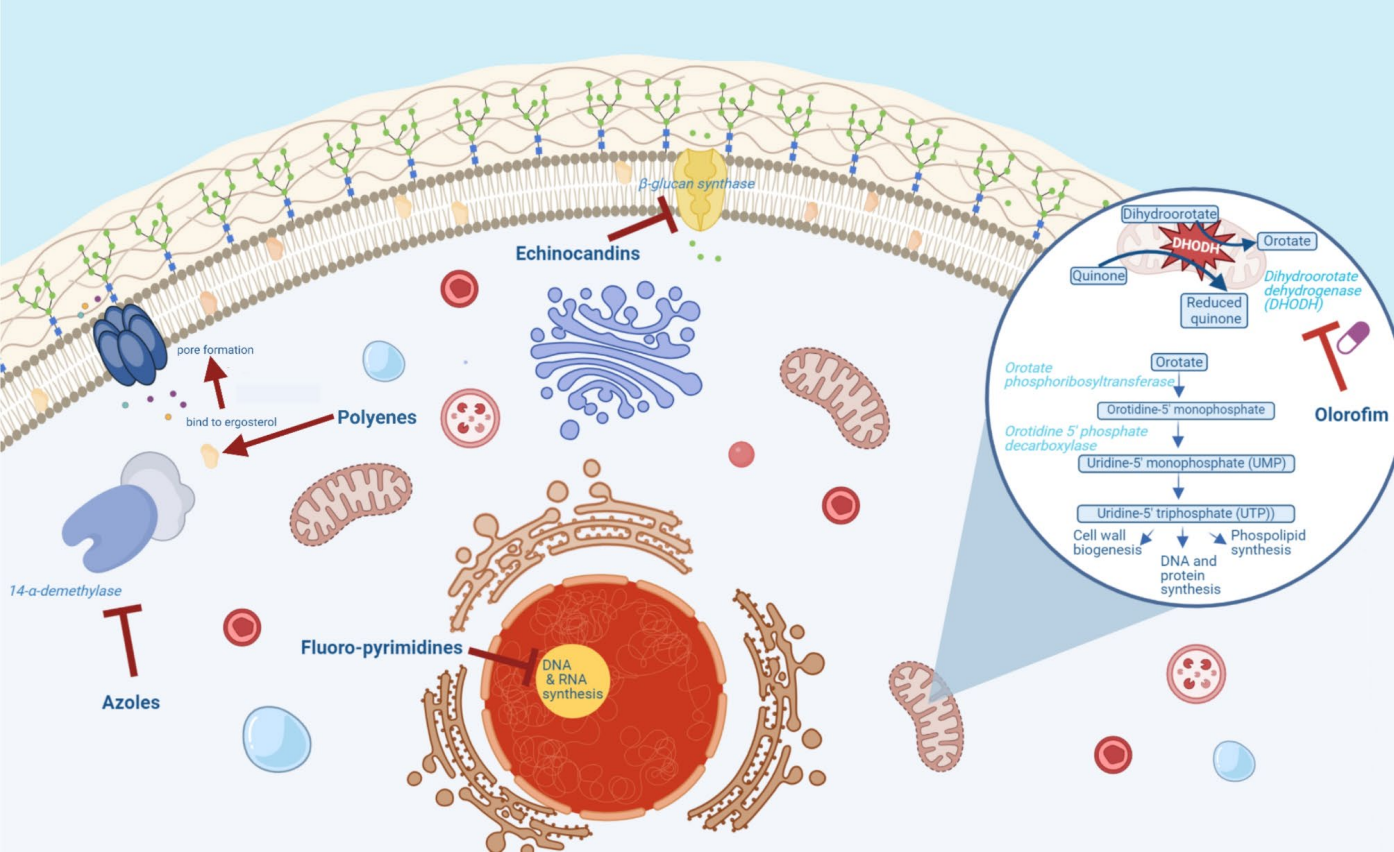
Current antifungal arsenal and mechanism of action of olorofim (Figure created with BioRender.com)

Currently, there are four classes of antifungals used in clinical practice for the treatment of IFDs. These classes include (a) the polyenes, such as liposomal amphotericin B (AmB), (b) the triazoles, such as fluconazole, itraconazole, voriconazole, posaconazole, and isavuconazole, (c) the echinocandins, such as caspofungin, anidulafungin and micafungin and (d) the antimetabolites, such as 5-fluorocytosine [29, 37–43]. A schematic overview of their mechanism of action is shown in Fig. 1. Liposomal AmB is a widely employed antifungal agent, serving as the primary treatment option for select IFDs, including mucormycosis and cryptococcosis. However, the use of liposomal AmB remains constrained by its associated toxicity and the exclusive parenteral administration route, which is shared with the echinocandins, another class of antifungal drugs with diminished efficacy against *Aspergillus* species [44, 45]. The triazoles show important drug-drug interactions (posaconazole, voriconazole and itraconazole in particular) and hepatotoxicity [46, 47]. Moreover, there is growing concern for antifungal resistance to current available azole antifungals, mainly driven by environmental fungicide use or long-term exposure to antifungals, in the setting of mould-active prophylaxis or as treatment of chronic antifungal infections, such as chronic pulmonary aspergillosis (CPA) [1–4, 48–54]. Azole resistance is associated with high overall mortality in patients with IA [3, 50, 52, 55].

In conclusion, there is a growing need for novel antifungals, considering spectrum of activity including resistance, toxicity, drug-drug interactions, and mode of administration. New antifungal drugs in various stages of clinical development include fosmanogepix (Gwt1 enzyme inhibitor), ibrexafungerp (triterpenoid), opelconazole (azole optimized for inhalation), rezafungin (echinocandin with long half-life time), AM2-19/SF001 (renal sparing polyene) [56], MAT2203 (oral encochleated amphotericin B) [57] and olorofim (dihydroorotate dehydrogenase inhibitor) [58].

Olorofim is a newly developed antifungal of the novel orotomide drug class, targeting the fungal dihydroorotate dehydrogenase (DHODH). It has been developed by F2G Biotech GmbH (Manchester, England) and is currently being tested in phase 3 trial (NCT05101187). It has been granted either orphan drug designation, qualified infectious disease product designation or breakthrough therapy designation by the European Medicines Agency (EMA) and Food and Drug Administration (FDA) for the treatment of various invasive fungal infections. The EMA and FDA approvals are summarized in Table 1.

**Table 1:** EMA and FDA approvals of olorofim. EMA (European Medicines Agency), FDA (Food and Drug Administration), CNS (central nervous system)

**FDA orphan drug designation**		
Invasive aspergillosis, lomentosporiosis, scedosporiosis	March	2020
Coccidiomycosis	June	2020
**FDA qualified infectious disease product designation**		
Invasive aspergillosis, coccidiomycosis, lomentosporiosis, scedosporiosis, scopulariopsis, fusariosis.	June	2020
**FDA breakthrough therapy designation**		
Invasive aspergillosis, lomentosporiosis, scedosporiosis, scopulariosis	November	2019
CNS coccidiomycosis	October	2020
**EMA orphan drug designation**		
Scedoscoporiosis	March	2016
Invasive aspergillosis	October	2016
Invasive scopulariopsis	January	2022

## Mechanism of action

Olorofim has been identified through a screen of a potential-drug library containing over 340.000 small molecules for in vitro activity against *Aspergillus fumigatus* [59].

Dihydroorotate dehydrogenase (DHODH) is the only oxidoreductase among the six enzymes catalyzing the pyrimidine biosynthesis and catalyzes the fourth step in the pyrimidine biosynthesis pathway, the conversion of dihydroorotate to orotate [59, 60]. Pyrimidines are essential for DNA and RNA synthesis and form lipid and carbohydrate metabolism precursors. Two classes of DHODH are identified, class I and II, based on amino acid sequence, subcellular location, and substrate preference. Most pathogens have class II DHODH which binds to the inner membrane of the mitochondria. It contains an N-terminal helix domain and a C-terminal domain. The N-terminal domain folds into two alpha-helices that form a channel to the active site and is the binding site for inhibitors of class II DHODH [61, 62].

Olorofim acts as a reversible DHODH inhibitor and inhibits DHODH by binding to the N-terminal helical domain of DHODH in *A. fumigatus*. The orotomides bind in the aforementioned channel where ubiquinone enters the enzyme from the inner mitochondrial membrane preventing reoxidation of the dihydroflavin mononucleotide (FMNH2) cofactor essential for the reaction to proceed. Human DHODH is only ca. 30% identical to its fungal homolog and is inhibited 2000-fold less effectively by olorofim [59]. This inhibition disrupts the formation of uridine-5’-monophosphate (UMP) and uridine-5’ triphosphate (UTP), essential precursors for cellular processes. UTP is particularly vital for the biosynthesis of UDP-sugars, serving as substrates for chitin synthetase and 1,3-β-D-glucan synthase, pivotal enzymes responsible for the synthesis of the fungal cell wall components chitin and 1,3-β-D-glucan, respectively. UMP and UTP are also important for production of cytosine, thymine, and uracil, and also in cell cycle regulation [63].

An in vitro study showed that conidia of *A.* *fumigatus* treated with olorofim did not germinate but isotropic growth continued. This supports the view that de novo pyrimidine synthesis is not required for isotropic growth but is needed for germination. Moreover, olorofim also inhibits polarized hyphal growth of *A. fumigatus in vivo*. Prolonged exposure to olorofim leads to inhibition of polarized hyphal growth, swelling and lysis. Thus, olorofim kills *A.* *fumigatus* in a time-dependent manner, with prolonged exposure leading to hyphal lysis (34 h) and leading to cell death after 120 h. Even after shorter exposures hyphae appear to recover poorly [64]. It is known that proliferating cells require active de novo pyrimidine biosynthesis [65, 66]. These observations support the hypothesis that de novo synthesis of pyrimidines is not required for conidial isotropic growth, but that it is vital for germination. Additionally, it seems that the presence of sufficient pyrimidines is necessary for polarized hyphal growth [64]. In addition to de novo synthesis, fungi are also able to acquire pyrimidine from the environment. In vitro susceptibility assays show that addition of exogenous pyrimidine reverses the activity of olorofim, but this only occurred at pyrimidine concentrations ≥ 5mM. Those concentrations are much higher than the concentration found within human serum (ca. 15µM). Hence, the scavenged pyrimidine by fungi from serum would not be sufficient to reverse the effect of olorofim in humans [59].

Vacuoles are important contributors to cell size and play a role in cell cycle regulation [67, 68]. Another in vitro study showed that vacuoles in hyphae exposed to olorofim significantly increased in size [69]. The enlargement of vacuoles may be related to cell cycle arrest, as cytoplasmic volume may be an important trigger for the G1 cell cycle phase in which mRNA and proteins are synthesized in preparation for mitosis. Large vacuoles are formed under nutrient-limited conditions in order to decrease cytoplasmic volume, and this decreases the need for nutrients and protein synthesis [68]. It is hypothesized that the formation of large vacuoles could be a sign of activation of autophagy [69].

Finally, treatment with olorofim leads to increased septation and cell wall remodeling with a decrease of beta-1-3-glucan at the hyphal tips and increased chitin content throughout the mycelium [69]. This may be due to a compensatory mechanism that is already known to occur with reduced 1,3-β-d-glucan levels following echinocandin exposure in different fungal species [70–72].

## Spectrum of activity

**Fig. 2:**
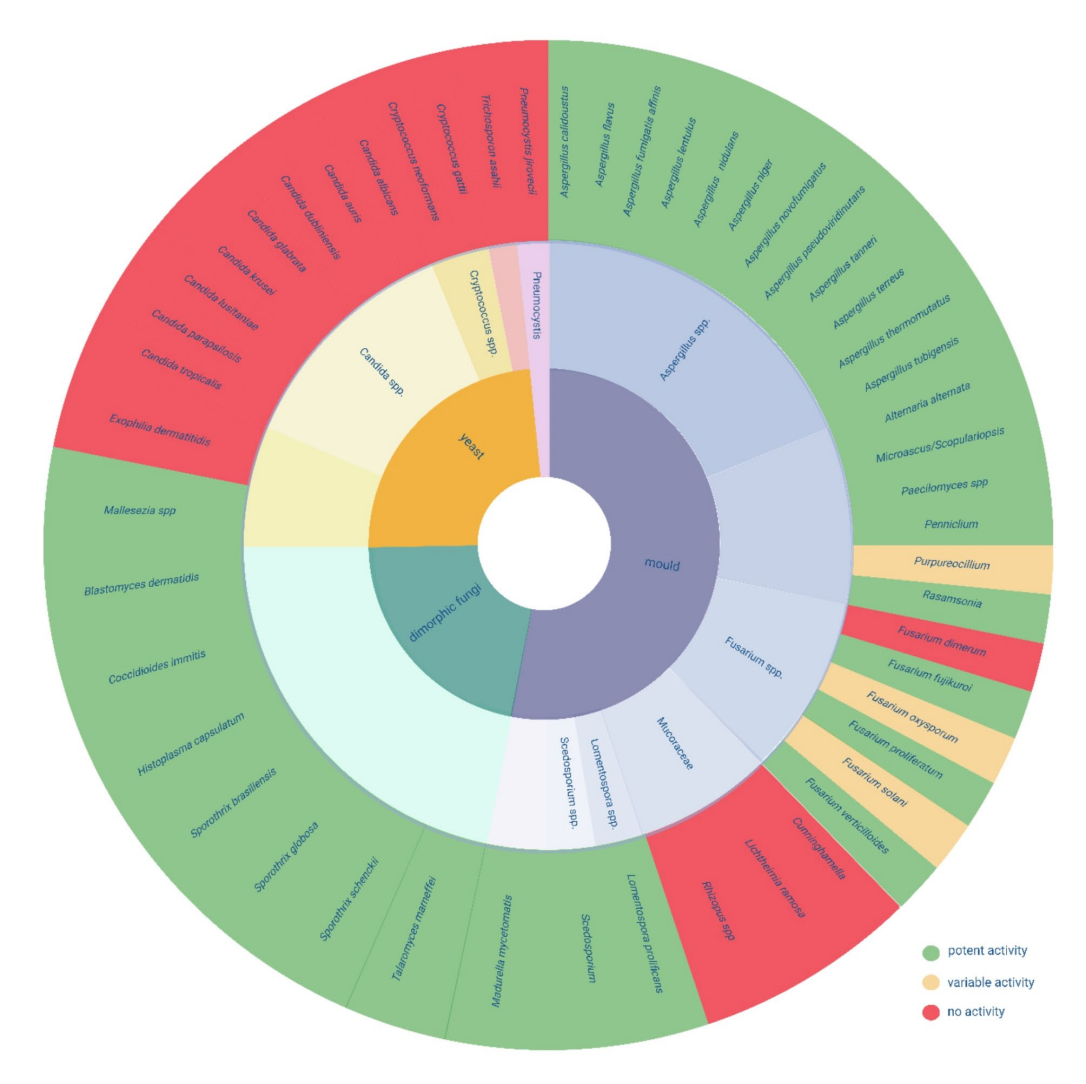
Spectrum of olorofim. (figure created with BioRender.com)

Olorofim shows a unique spectrum of activity. *A schematic overview is shown in* Fig. 2. This unique spectrum of activity of olorofim has been attributed to differences in the DHODH enzymes among various groups of fungi [59]. Despite their classification as class II DHODH enzymes, those derived from *Candida* and *Cryptococcus* species exhibit a more distant relationship to the DHODH of fungi susceptible to inhibition by olorofim [59]. *Mucorales* species have only DHODH class IA, and thus lack DHODH class II which is the target of olorofim. Some of the dematiaceous mould species show mixed susceptibility to olorofim, probably because they harbor dihydrouracil oxidases rather than DHODHs [73]. Olorofim exhibits no in vitro activity against yeasts, including *Candida* species [70] and *Cryptococcus* species [74], *Mucorales* species, thermally monomorphic molds, *Alternaria alternata* [75] and *Exophilia dermatitidis* [76].

### In vitro

Olorofim demonstrates activity against several fungi that either demonstrate resistance or have reduced susceptibility against current available antifungals. It shows good in vitro activity against all *Aspergillus* species, including cryptic species and azole or AmB resistant isolates, e.g. *A. lentulus*,* A. fumigati affinis*,* A. novofumigatus*,* A. thermomutatus*,* A. calidoustus*,* A. flavus*,* A. nidulans*,* A. tubigensis*,* A. terreus*,* A. udagawae*,* A. fumisynnematus*,* A. tanneri*,* A. pseudoviridinutans*,* A. versicolor* and others [51, 64, 70, 75–89]. Olorofim even shows significant inhibitory activity at early-stage growth of A. *fumigatus*, *A. flavus* and *A. niger* at > 100.000-fold sub-MIC drug concentrations [90]. It is active against endemic mycoses, such as *Coccidioides immitis*,* Histoplasma capsulatum*,* Blastomyces dermatitidis* and *Sporothrix (brasiliensis*,* globosa*,* schenckii)* species [59, 91–93]. The drug also shows activity against *Lomentospora prolificans* and *Scedosporium* species [59, 76, 77, 94–98]. *Fusarium species* show variable susceptibility to olorofim with no activity against *F. dimerum*, but showing good activity against *F. verticilloides*,* F. fujikuroi* and *F. proliferatum* and variable activity against *F. solani* and *F. oxysporum* [49, 74, 77, 78, 99–101]. Moreover, there is in vitro activity against *Microascus/Scopulariopsis*,* Penicillium*,* Paecilomyces*,* Purpereocillium*,* Rasamsonia*,* Talaromyces*,* Trichophyton* (including *T. indotineae* and other terbinafine-resistant isolates [75]) and *Madurella mycetomatis*, the most common cause of eumycotic mycetoma [59, 70, 75–78, 102–106]. Interestingly, olorofim also exhibits activity against biofilms, as shown in vitro for *Aspergillus fumigatus* and *Lomentospora prolificans* [95, 107].

### In vivo

The in vivo activity of olorofim has been evaluated in several murine models of aspergillosis, coccidioidomycosis, lomentosporiosis and scedosporiosis.

In a neutropenic murine model of invasive pulmonary aspergillosis (IPA), mice were infected with a well-characterized *A. fumigatus* strain (NIH 4215). Survival was significantly improved by treatment with olorofim, even in strains with azole resistance due to CYP51A mutations [59]. In another neutropenic murine model of IPA, treatment with olorofim improved survival significantly in mice infected with either azole-susceptible or azole-resistant (TR34/L98H- and G138C-mutated) *A. fumigatus* isolates, and a significant dose-dependent reduction of serum galactomannan (GM) was observed in comparison to those treated with a humanized dose of posaconazole [81]. Similar results were shown in a murine model of sinopulmonary aspergillosis with *A. flavus*, where olorofim showed a concentration-dependent decline in GM and showed increased survival, greater than for posaconazole. Dose-enhanced histopathological clearance of fungi from the lung tissue was also observed [80]. Another murine model of neutropenic mice of disseminated aspergillosis with *Aspergillus terreus* was conducted. Olorofim showed prolonged survival in these mice, superior to AmB and resulted in a decreased histopathological fungal burden in kidney tissue [82]. In murine models of invasive aspergillosis in neutropenic CD-1 mice and mice with chronic granulomatous disease (gp−/− phox mice) infected with *A. fumigatus*,* A. nidulans*,* or A. tanneri*, treatment with intraperitoneal olorofim resulted in improved survival, reduction in GM levels and lower fungal burden, measured by quantitative PCR (DNA) and through histopathology, irrespective of the azole susceptibility of the *Aspergillus* species. Less than 10% of the mice in the control group survived for 10 days [108].

For central nervous system (CNS) coccidioidomycosis, a murine model was performed in which arthroconidia of *C. immitis* were inoculated intracranially. Olorofim showed significantly improved survival and reduced brain fungal burden compared to controls, as measured by colony-forming units, both in a time-dependent manner. Both survival and reductions in brain fungal burden were enhanced when the olorofim dosing frequency was increased from twice daily to three times daily despite no changes in the overall daily doses [93].

Lastly, in a murine model of neutropenic cyclophosphamide-immunosuppressed CD-1 mice, the mice were infected by *Scedosporium apiospermum*, *Pseudallescheria boydii (Scedosporium boydii)* and *Lomentospora prolificans* and treated with intraperitoneal olorofim. Treatment with olorofim significantly improved survival as compared to controls. The levels of beta-D-glucan (BDG) and the fungal DNA burden were significantly suppressed. This was histopathologically confirmed as the kidneys of the treated mice showed no or only a few lesions with hyphal elements [109].

## Pharmacokinetics and pharmacodynamics

Thirteen phase I clinical trials of olorofim have been completed. Safety, tolerability, pharmacokinetics (PK), and pharmacodynamics (PD) have been assessed for single and multiple doses of intravenous (IV) and oral formulations (NCT02808741, NCT02737371, NCT02342574, NCT02394483, NCT02142153, NCT02680808, NCT02730442, NCT03340597, NCT04171739, NCT04039880, NCT04752540, NCT04207957, NCT05200286) [64, 69, 79, 82, 102, 110–112].

Olorofim can be administered intravenously and orally, although the majority of studies have focused on the oral formulation. Pharmacokinetics have been reported from studies in healthy volunteers following both routes of administration. Due to its insolubility in water, the IV formulation of olorofim uses a beta-hydroxypropyl cyclodextrin vehicle [102].

Table 2 provides an overview of the pharmacological properties of olorofim based on PK studies across various animal models and in healthy human volunteers. The studies assessed its bioavailability, tissue distribution, dosing regimens and the effects of food on its pharmacokinetics. Key findings indicate that olorofim has significant oral bioavailability, high protein binding and the ability to penetrate the blood-brain barrier, suggesting its potential for treating central nervous system (CNS) fungal infections [111–119].

**Table 2:** Pharmacological properties of olorofim. PO (oral), IV (intravenous), CNS (central nervous system), MIC (minimal inhibitory concentration), C_min_ (trough concentration)

Pharmacological properties of olorofim			
Parameter	Main findings	Studied species	Study information
**Oral bioavailability**	45%-82%, administration irrespective of food administration	Rats, Mice, Cynomolgus Monkeys Healthy Male and Female Volunteer	Single oral and IV dosing (mg/kg) Open-label study with fed and fasted conditions
**C**_**max**_	3.26µg/ml	Healthy male volunteers	Single IV administration 4mg/kg over 4h
	2.21 µg/ml (1.50–3.23 µg/ml)	Healthy volunteers	Multi-dose oral
	1.66µg/ml (0.53–3.75µg/ml)	Patients with IFD	Multi-dose oral
**AUC**_0 − inf_	40.94 µg.h/mL	Healthy male volunteers	Single IV administration 4mg/kg over 4h
**AUC**_**0 − 24h**_	23.8µg.h/ml (16.6–31.4µg.h/ml)	Healthy volunteers	Multi-dose oral
**AUC**_**0 − 24h**_	20.7µg.h/ml (7.59–52.5µg.h/ml)	Patients with IFD	Multi-dose oral
**T**_**1/2**_	24-30h	Healthy male volunteers	Single IV administration 4mg/kg over 4h
**Plasma protein binding**	99.7%	Rats, Mice, Cynomolgus Monkeys	Single oral and IV dosing ( mg/kg)
**V**_**d**_	2.89-3.49L/kg + CNS distribution	Healthy male volunteers	Single IV administration 4mg/kg over 4h
**Administration via nasogastric tube**	Similar systemic exposure, C_max_ 91.44%, AUC 87.62% compared to oral	Healthy male and Female Volunteers	Open-label study comparing administration methods
**Enterohepatic recirculation**	Secondary peaks observed, suggesting enterohepatic recirculation	Healthy male volunteers	Multi-dose IV and oral dosing studies
**Blood-brain barrier crossing**	Potential for CNS penetration (mean brain ratio of 1:1)	Rats	Single 2-hour IV infusion of ^14^C-olorofim (10mg/kg)
**Dosing**	- **PO**: 150 mg BID on day 1, then 90 mg BID - **IV**: no standard dose		
**Metabolism and elimination**	Hepatic metabolism		
**Drug-drug interactions**	Weak CYP3A4 inhibitor, CYP3A4 substrate		
**PK/PD Target**	C_min_/MIC		
**Adverse events**	- **PO**: mild gastrointestinal intolerance - **IV**: infusion reactions		

### Drug-drug interactions

As olorofim is metabolized by CYP3A4, it is vulnerable to drug-drug interactions [120–122]. In an open-label study, healthy male volunteers received single IV dose of olorofim on days 1 and 8, with oral fluconazole (a moderate CYP3A4 and CYP2C9 inhibitor) being given on days 4 to 8 (loading dose of 800 mg on day 4, followed by 400 mg OD). PK sampling for olorofim was performed 72 h after each dose of olorofim. The systemic exposure to olorofim (based on AUC_0 − 72 h_) was 1.5 to 1.6 times higher when administered in the presence of fluconazole than when administered alone and no significant increase in C_max_ was observed [121]. Another open-label study evaluated the effect of itraconazole (a potent CYP3A4 inhibitor) and rifampicin (a potent CYP3A4 inducer) on the pharmacokinetics of a single oral dose of olorofim. Healthy male and female volunteers were divided into two cohorts. In the first cohort the volunteers received a single oral dose of olorofim (60 mg) on days 1 and 11, in combination with 200 mg itraconazole OD on days 6 to 15. PK sampling for olorofim was performed for 120 h after each dose of olorofim. Systemic exposure to olorofim (based on mean C_max_ and mean AUC_0 − 120 h)_ increased by 240% and 152%, when given in combination with itraconazole. In the second cohort, subjects received a single oral dose of olorofim (120 mg) on days 1 and 11, in combination with 600 mg rifampicin OD on days 6 to 15. PK sampling for olorofim was performed for 120 h after each dose of olorofim. Mean olorofim plasma concentrations were lower and mean C_max_ and mean AUC_0 − 120 h_ decreased with 55.72% and 26.11%, respectively [122].

Olorofim appears to be a weak CYP3A4 inhibitor. In an open-label study in healthy volunteers, an increase in midazolam concentrations was observed when this benzodiazepine was administered on day seven compared one prior to the start of a seven-day course of olorofim, as evidenced by an increase in mean midazolam concentration from 1.27 µg/mL on day one to 1.65 µg/mL on day seven [120].

In addition, in an open-label phase IIB salvage study (NCT03583164), solid organ transplant (SOT) recipients received olorofim for the treatment of severe IFDs. In these patients, drug-drug interactions were predictable and easy to manage. Small reductions in sirolimus and tacrolimus (both CYP3A4 substrates) were sometimes required due to the (weak) inhibition of CYP3A4 by olorofim and were managed with standard TDM of the calcineurin inhibitors [123].

The need for Therapeutic Drug Monitoring (TDM) in patients with invasive mould infections with limited or no treatment options was evaluated in a phase 2b study. Geometric mean steady-state pharmacokinetic parameters of olorofim were similar between the TDM and fixed-dosed groups (*n* = 90). Mean olorofim pre-dose concentrations were consistent over time for all patients, regardless of the group and regimen. In addition, C_min_ exposures consistently exceeded the pharmacodynamic target (C_min_≥0.2 µg/ml) in both groups. Thus, when administered as a standard dose (loading dose of 150 mg BID on day 1, followed by 90 mg BID ) adequate exposure is observed in the populations studied in this trial and may not need to be confirmed by TDM [114, 119, 123].

### In vivo efficacy and pharmacodynamics

In a neutropenic murine model of IPA survival and reductions in serum GM were enhanced with more frequent dosing and dose-fractionation experiments demonstrated time-dependent activity^65^. Similar results were shown in a murine model of sinopulmonary aspergillosis with *A. flavus*, where olorofim showed a concentration-dependent decline in GM and showed increased survival, greater than for posaconazole. Dose-enhanced histopathological clearance of fungi from the lung tissue was also observed [80]. Moreover, in a mouse model of CNS infections with *C. immitis* olorofim showed significantly improved survival and reduced brain fungal burden, as measured by colony-forming units, both in a time-dependent manner [93]. Both survival and reductions in brain fungal burden were enhanced when the olorofim dosing frequency was increased from twice daily to three times daily despite no changes in the overall daily doses. These results agree with the time-dependent antifungal activity with C_min_/MIC being the pharmacokinetic/pharmacodynamic (PK/PD) parameter most associated with in vivo efficacy [81]. This is consistent with the time-dependent activity described in vitro. Interestingly, the effects of olorofim in vitro change from fungistatic to fungicidal with prolonged exposure [64].

## Safety

Olorofim was well tolerated in phase I trials. When administered IV adverse events were mild or moderate. Infusion-related reactions, such as phlebitis (39%), infusion site pain (44%), and dizziness (67%) were the most commonly reported adverse events [117].

Oral olorofim was well-tolerated in the FORMULA-OLS/study 32(NCT03583164) trial, even in the extended treatment arm with patients exposed to > 2 years on treatment. Changes in liver biochemistry at least possibly related to olorofim occurred in 9.9% (as judged by an independent hepatic advisory committee) and were managed by dose reduction or discontinuation. Permanent discontinuation was needed in 2.5%. Mild gastrointestinal intolerance occurred in 9.9% [124].

### Resistance

Azole-resistant aspergillosis is thought to be due to inhalation of resistant conidia that have developed resistance due to long-term exposure to azole fungicides in the environment [2]. However, acquired resistance due to azole exposure in patients has also been reported [77, 125–128].

Spontaneous olorofim mutation occurs at a negligible frequency of 1.3 × 10^− 7^ to 6.9 × 10^− 9^ [125]. No resistance was detected in 1.423 mold isolates (including *Aspergillus* and *Scedosporium*) [77]. This was confirmed in a screening of 975 clinical isolates of *Aspergillus fumigatus*, where no intrinsic resistance was detected and no cross-resistance to azoles was detected [125].

Olorofim resistance (MIC > 8 mg/L) can develop secondary due to mutations within the gene encoding for DHODH, the *PyrE* gene, resulting in various amino acid substitutions with a hotspot at the G119 locus at the entrance to the active site of DHODH. Consequently, the mutant DHODH demonstrates reduced affinity for olorofim resulting in high levels of resistance. These mutations had a small but significant negative effect on the growth rate of these mutant strains [77, 125]. Isolates exhibiting olorofim MICs of > 8 mg/l could be selected in laboratory settings by employing a high number of conidia and prolonged exposure to this antifungal agent [125]. Target sequencing revealed one alteration (Q36L) in a single isolate which is not of clinical relevance as it did not affect susceptibility to olorofim [77].

Treatment-induced resistance of olorofim has not been reported to date [125].

The aforementioned studies did not demonstrate cross-resistance between olorofim and the azoles. However, a unidirectional antagonistic effect of the triazoles on olorofim in vitro has recently been identified, due to azole-induced up-regulation of the pyrimidine biosynthetic pathway which is the target of olorofim. Loss of function of two transcription factors, HapB a member of the heterotrimeric HapB/C/E (CBC) complex and the regulator of nitrogen metabolism genes AreA, led to cross-resistance to both the azoles and olorofim. These data suggest that there is a complex crosstalk between the ergosterol and pyrimidine biosynthetic pathways. Moreover, the overexpression of any constituent within the pyrimidine biosynthetic pathway yielded a modest augmentation in the susceptibility of *A. fumigatus* to azoles. This suggests that certain strains that were resistant to olorofim may exhibit heightened susceptibility to azoles [110]. The clinical significance of these data remains to be determined.

There is an emerging concern that agrochemical fungicides may cause cross-resistance to *Aspergillus species* in humans [1]. Ipflufenoquin is an agrochemical fungicide that is a potent inhibitor of DHODH and therefore has the same mechanism of action as olorofim. In vitro exposure of *A. fumigatus* to ipflufenoquin can select for strains that are resistant to olorofim. Resistance is caused by non-synonymous SNPs in the *PyrE* gene that confer cross-resistance to olorofim which has the same target enzyme as ipflufenoquin. In addition, no fitness defect was observed in these resistant strains, suggesting that there is no barrier for these strains to survive and become dominant in the environment [53, 129].

### Clinical outcomes

Data from the open-label, single-arm, phase IIb FORMULA-OLS/Study 32 (NCT03583164) includes patients with IFDs due to *Lomentospora prolificans*, Scedosporium spp., Aspergillus spp. and other resistant fungi lacking suitable alternative treatment options. 202 patients were enrolled (modified intention to treat), having *Aspergillus* spp. (proven or probable IA) (101, including 22 cases with azole-resistant strains), *Lomentospora prolificans* [26], *Scedosporium* spp. [22], *Coccidioides* spp. [41], *Scopulariopsis* spp. [6] and other fungi such as *Fusarium or Madurella* spp. [8]. The overall success rate at day 42 and day 84 was 28.7% and 27.2%, and 34.7% and 33.7% respectively for the overall cohort and IA. The overall success rate in IFDs other than coccidiomycosis (*n* = 161) was 36.0% at day 42. All-cause mortality at day 42 and day 84 was 11.4% and 15.8% for the total cohort, and 17.8% and 25.7% for IA respectively. If stable disease was considered as success, which is certainly acceptable from a clinical point of view in these difficult-to-treat infections, then the success rate was 75.2% at day 42 and 63.4% at day 84 [130]. For *Coccidioides* spp. there was no response on day 42 and day 84, as this can only be evaluated by proven fungal eradication. However, clinical benefit was obtained in 75.6% and 73.2% at day 42 and day 84, respectively [124].

In addition to this phase IIb study, there is rather limited clinical data available from case reports and one case series. Three case reports showed positive clinical outcomes for patients with invasive lomentosporiosis. One case involved a 56-year-old woman with disseminated lomentosporiosis after receiving intensive chemotherapy for T-ALL who failed on voriconazole in combination with terbinafine and surgical debulking of the spine. Improvement was observed within 6 months after initiation of olorofim [131]. The second case referred to a 49-year-old woman with extensive lomentosporiosis of her right breast implant refractory to surgery, voriconazole, terbinafine, posaconazole, miltefosine and anidulafungin. After initiation of olorofim, gradual clinical improvement of the infection was observed [132]. The third case reports a 57-year old lung transplant recipient, under active immunosuppression, with a disseminated infection and endophthalmitis with *Lomentospora prolificans* who was systemically treated with voriconazole, terbinafine and micafungin. The patient was later started on olorofim, partly in combination with voriconazole and terbinafine, and initially responded well to therapy with possibility of discharge but eventually died due to progressive disease. Susceptibility testing for olorofim was not performed on the positive culture [133]. Another case report involves a 45-year-old man with disseminated coccidiomycosis, including infection of the CNS. Treatment with fluconazole, voriconazole, itraconazole, posaconazole and micafungin failed. Then combination therapy of posaconazole and olorofim was started with subsequent rapid clinical improvement and decline of the complement fixation titer [134]. Olorofim has also reported to be successful in a 14-year-old male patient with IPA and underlying X-linked chronic granulomatous disease. Fungal culture of lung biopsy showed *A. fumigatus* with multi-azole resistance due to a mutation in the CYP51A gene. The patient was refractory despite treatment with voriconazole and caspofungin. Due to the resistance, liposomal AmB was started with partial regression of the IPA. However, due to nephrotoxicity the AmB was discontinued, and surgery was performed. olorofim was started (initially in combination with caspofungin), resulting in a complete and long-lasting remission [135]. Finally, a case series describes three patients with refractory *Microascus* spp. bronchopulmonary infection who were treated with olorofim. These included a 17-year-old boy in a polytrauma setting who was successfully treated with a combination of olorofim and terbinafine for a pulmonary infection with *M. melanosporus*. The two remaining patients were lung transplant recipients who were successfully treated with olorofim (for the latter in combination with terbinafine) for pulmonary infection with *M. cirrosus* [136].

## Ongoing clinical trials

Olorofim is currently in a phase 3, adjudicator-blinded, randomized trial (NCT05101187 – registration date 7th of September 2021, OASIS study, sponsored by F2G Biotech GmbH) which evaluates the efficacy and safety of treatment with oral olorofim versus treatment with liposomal AmB followed by standard of care in patients with proven or probable IA. Primary outcome is all-cause mortality at treatment day 42. Secondary endpoints include adjudicated assessment of overall outcome at day 42, day 84 and end of treatment; investigator-assessed overall response and safety.

### Opinion

Despite considerable improvements in treatment and diagnostics of invasive fungal diseases over the past two decades, these infections remain devastating diseases for the ever-growing population of immunocompromised patients. The four currently available antifungal drug classes (azoles, polyenes, echinocandins and anti-metabolites) are limited by one or more of the requirements for intravenous administration, clinically significant drug-drug interactions, therapeutic drug monitoring, and frequent adverse events. Further, the worldwide emergence of triazole-resistant *Aspergillus *species is a concern, whereas *Scedosporium*, *Lomentospora*, and *Fusarium* species are resistant to multiple if not all antifungal agents. Olorofim is one of the few new antifungal drugs in late-stage clinical development that targets some of the critical members of the WHO fungal pathogen priority list. The drug not only provides a treatment option for patients lacking suitable treatment options, but also alleviate treatment in outpatient settings for prolonged periods of time (as evidenced in the open-label phase IIb study). However, pending the results of the ongoing, randomized phase 3 study, supplemental data are needed, including pharmacokinetic profiling in special populations (e.g., obese patients, ICU populations, .), pediatric data, dosing adaptations (if any) in patients with severe renal and hepatic dysfunctions, extended drug-drug interaction profiling and need for therapeutic drug monitoring.

## Conclusion

Olorofim is a novel antifungal with promising activity against difficult to treat IFDs with little or no therapeutic options, such as azole-resistant aspergillosis, breakthrough infections, scedosporiosis, lomentosporiosis and invasive scopulariopsis infections, for which it shows very low MICs and biofilm activity. It has a potential role in the treatment of some endemic mycoses, such as coccidioidomycosis, talaromycosis and mycetoma. It shows excellent tissue distribution in the lung, liver and kidney and shows good CNS penetration. Its major limitations are its rather narrow spectrum of activity, lacking activity against yeasts, including *Candida* spp. and *Cryptococcus* spp., and against *Mucorales*. It is well-tolerated, even during extended treatment. As it is orally bio-available it might be suitable for the long-term treatment of chronic and allergic fungal diseases. It shows no cross-resistance with current available antifungals. Olorofim is metabolized by CYP3A4 and a weak CYP3A4 inhibitor, making it vulnerable to drug-drug interactions, but these seem to be predictable and readily manageable. Recent clinical data is promising, but more data is needed to define its place more clearly as a novel agent in the antifungal arsenal. Also, implementation of stewardship programs and epidemiologic surveillance, will be necessary to monitor and reduce resistance development, while further ensuring the safety and efficacy of this novel agent.

## Electronic supplementary material

Below is the link to the electronic supplementary material.

Supplementary Material 1

Supplementary Material 2

## Data Availability

All data from this review comes from a systematic search of the literature in PubMed, using the terms ‘olorofim’ and ‘F901318’. All studies are included in this scoping review and are cited and available in the references.

## Abbreviations

ALL: Acute lymphocytic leukemia
AmB: Amphotericin B
AUC: Area under the curve
BDG: beta-D-glucan
BID: Twice a day
C_max_: Peak concentration
C_min_: Trough concentration
CNS: Central nervous system
COPD: Chronic obstructive pulmonary disease
CPA: Chronic pulmonary Aspergillosis
CYP: Cytochromes
DHODH: Dihydroorotate dehydrogenase
DNA: Deoxyribonucleic acid
EMA: European Medicines Agency
FDA: Food and Drug Administration
FMNH2: Dihydroflavin mononucleotide
GM: Galactomannan
GVHD: Graft-versus-host disease
HSCT: Hematopoietic stem cell transplant
IA: Invasive aspergillosis
IFD: Invasive fungal disease
IPA: Invasive pulmonary aspergillosis
IV: Intravenous
MIC: Minimal inhibitory concentration
OD: Once daily
PD: Pharmacodynamics
PK: Pharmacokinetics
PO: Oral
RNA: Ribonucleic acid
SNPs: Small nucleotide polymorphisms
SOT: Solid organ transplant
TDM: Therapeutic Drug Monitoring
T_1/2_: Half-life
UMP: Uridine-5’-monophosphate
UTP: Uridine-5’ triphosphate
V_d_: Volume of distribution
WHO: World Health Organization
