# Correction: Review of the novel antifungal drug olorofim (F901318)

**DOI:** 10.1186/s12879-024-10295-2

**Published:** 2024-12-12

**Authors:** Yuri Vanbiervliet, Tine Van Nieuwenhuyse, Robina Aerts, Katrien Lagrou, Isabel Spriet, Johan Maertens

**Affiliations:** 1 Department of Haematology, Department of Microbiology, Immunology and Transplantation, University Hospitals Leuven, KU Leuven, Herestraat 49, Leuven, 3000 Belgium; 2 Pharmacy Department, University Hospitals Leuven, Herestraat 49, Louvain, 3000 Belgium; 3 Department of Laboratory Medicine and National Reference Center for Mycosis, Department of Microbiology, Immunology and Transplantation, University Hospitals Leuven, KU Leuven, Herestraat 49, Leuven, 3000 Belgium; 4 Department of Pharmaceutical and Pharmacological Sciences, Pharmacy Department, University Hospitals Leuven, KU Leuven, Herestraat 49, Leuven, 3000 Belgium

**Correction: BMC Infect Dis 24, 1256 (2024)**

**https://doi.org/10.1186/s12879-024–10143-3**

Following publication of the original article [1], the authors identified **an error in **Table 2 specifically in the AUC-values and C_max_ values, which were mistakenly mixed up between two studies during the final compilation of the data. While this does not change the overall conclusions of the paper, the authors believe it is important to correct the record to maintain the integrity of the research.

Originally published Table 2:

**Table 2:** Pharmacological properties of olorofim. PO (oral), IV (intravenous), CNS (central nervous system), MIC (minimal inhibitory concentration), C_min_ (trough concentration)

Pharmacological properties of olorofim			
**Parameter**	**Main findings**	**Studied species**	**Study information**
**Oral bioavailability**	45–82%, administration irrespective of food administration	Rats, Mice, Cynomolgus Monkeys Healthy Male and Female Volunteer	Single oral and IV dosing (mg/kg) Open-label study with fed and fasted conditions
**C**_**max**_	2.2 µg/mL	Healthy male volunteers	Single IV administration 4 mg/kg over 4 h
**AUC**_0−inf_	11.9 µg.h/mL (1.31–40.94 µg.h/mL)	Healthy male volunteers	Single IV administration 4 mg/kg over 4 h
**T**_**1/2**_	24–30 h	Healthy male volunteers	Single IV administration 4 mg/kg over 4 h
**Plasma protein binding**	99.7%	Rats, Mice, Cynomolgus Monkeys	Single oral and IV dosing ( mg/kg)
**V**_**d**_	2.89–3.49 L/kg + CNS distribution	Healthy male volunteers	Single IV administration 4 mg/kg over 4 h
**Administration via nasogastric tube**	Similar systemic exposure, C_max_ 91.44%, AUC 87.62% compared to oral	Healthy male and Female Volunteers	Open-label study comparing administration methods
**Enterohepatic recirculation**	Secondary peaks observed, suggesting enterohepatic recirculation	Healthy male volunteers	Multi-dose IV and oral dosing studies
**Blood–brain barrier crossing**	Potential for CNS penetration (mean brain ratio of 1:1)	Rats	Single 2-h IV infusion of ^14^C-olorofim (10 mg/kg)
**Dosing**	- PO: 150 mg BID on day 1, then 90 mg BID - IV: no standard dose		
**Metabolism and elimination**	Hepatic metabolism		
**Drug-drug interactions**	Weak CYP3A4 inhibitor, CYP3A4 substrate		
**PK/PD Target**	C_min_/MIC		
**Adverse events**	- PO: mild gastrointestinal intolerance - IV: infusion reactions		

Corrected Table 2:

**Table 2:** Pharmacological properties of olorofim.PO (oral), IV (intravenous), CNS (central nervous system), MIC (minimal inhibitory concentration), C_min_ (trough concentration)

Pharmacological properties of olorofim			
**Parameter**	**Main findings**	**Studied species**	**Study information**
**Oral bioavailability**	45%−82%, administration irrespective of food administration	Rats, Mice, Cynomolgus Monkeys Healthy Male and Female Volunteer	Single oral and IV dosing (mg/kg) Open-label study with fed and fasted conditions
**C**_**max**_	3.26 µg/ml	Healthy male volunteers	Single IV administration 4 mg/kg over 4 h
	2.21 µg/ml (1.50–3.23 µg/ml)	Healthy volunteers	Multi-dose oral
	1.66 µg/ml (0.53–3.75 µg/ml)	Patients with IFD	Multi-dose oral
**AUC**_0-inf_	40.94 µg.h/mL	Healthy male volunteers	Single IV administration 4 mg/kg over 4 h
**AUC**_**0–24 h**_	23.8 µg.h/ml (16.6–31.4 µg.h/ml)	Healthy volunteers	Multi-dose oral
**AUC**_**0–24 h**_	20.7 µg.h/ml (7.59–52.5 µg.h/ml)	Patients with IFD	Multi-dose oral
**T**_**1/2**_	24–30 h	Healthy male volunteers	Single IV administration 4 mg/kg over 4 h
**Plasma protein binding**	99.7%	Rats, Mice, Cynomolgus Monkeys	Single oral and IV dosing ( mg/kg)
**V**_**d**_	2.89–3.49L/kg + CNS distribution	Healthy male volunteers	Single IV administration 4 mg/kg over 4 h
**Administration via nasogastric tube**	Similar systemic exposure, C_max_ 91.44%, AUC 87.62% compared to oral	Healthy male and Female Volunteers	Open-label study comparing administration methods
**Enterohepatic recirculation**	Secondary peaks observed, suggesting enterohepatic recirculation	Healthy male volunteers	Multi-dose IV and oral dosing studies
**Blood–brain barrier crossing**	Potential for CNS penetration (mean brain ratio of 1:1)	Rats	Single 2-h IV infusion of ^14^C-olorofim (10 mg/kg)
**Dosing**	- **PO**: 150 mg BID on day 1, then 90 mg BID - **IV:** no standard dose		
**Metabolism and elimination**	Hepatic metabolism		
**Drug-drug interactions**	Weak CYP3A4 inhibitor, CYP3A4 substrate		
**PK/PD Target**	C_min_/MIC		
**Adverse events**	- **PO**: mild gastrointestinal intolerance - **IV**: infusion reactions		

The text referring to Table 2 (page 8, row 222) was also modified accordingly (added text in bold): “Table 2 provides an overview of the pharmacological properties of olorofim based on PK studies across various animal models and in healthy human volunteers **and patients with IFD**.”

The original article has been corrected.
