# Supplementary material for: Review of the novel antifungal drug olorofim (F901318)
Source: BMC Infect Dis. 2024 Nov 7;24:1256. doi: 10.1186/s12879-024-10143-3 (PMC11542455; doi:10.1186/s12879-024-10143-3)
Supplement: Supplementary file 1 — Supplementary Material 1 [file 12879_2024_10143_MOESM1_ESM.pdf]

Supporting information

Author information

Yuri Vanbiervliet:

- MD, Department of Haematology, University Hospitals Leuven and Department of Microbiology, Immunology and Transplantation, KU Leuven, Leuven, Belgium ORCID 0000-0002-6169-1920
- E-mail: yuri.vanbiervliet@uzleuven.be (corresponding author). UZ Leuven, Herestraat 49, 3000 Leuven, Belgium.

Tine Van Nieuwenhuyse:

- PharmD, Pharmacy Department; University Hospitals Leuven UZ Leuven, Belgium, ORCID 0000-0002-0898-904X
- E-mail: tine.vannieuwenhuyse@uzleuven.be (co-authorship)

Robina Aerts:

- MD Department of Haematology, University Hospitals Leuven and Department of Microbiology, Immunology and Transplantation, KU Leuven, Leuven, Belgium, ORCID 0000-0001-6337-6636
- E-mail: robina.aerts@kuleuven.be

Isabel Spriet:

- PharmD PhD Pharmacy Department University Hospitals Leuven and Dpt. Of Pharmaceutical and Pharmacological Sciences, KU Leuven, Belgium. ORCID: 0000-0001-6342-0676
- E-mail: isabel.spriet@uzleuven.be

Katrien Lagrou:

- PharmD, Department of Laboratory Medicine and National Reference Center for Mycosis, University Hospitals Leuven; Department of Microbiology, Immunology and Transplantation, KU Leuven, Leuven, Belgium. ORCID 0000-0001-8668-1350
- E-mail: katrien.lagrou@uzleuven.be

Johan Maertens:

- MD PhD Department of Haematology, University Hospitals Leuven and Department of Microbiology, Immunology and Transplantation, KU Leuven, Leuven, Belgium, ORCID 0000-0003-4257-5980
- Email: johan.maertens@uzleuven.be

Leuven, 3000, Belgium

24th of July 2024

Cover letter

Dear editor, dear Dr. N. Hirst, dear editorial board,

I am writing to submit our manuscript entitled '**Review of the novel antifungal drug olorofim (F901318)**'. In this manuscript we present a scoping review concerning olorofim, the first-in class molecule of a novel class of antifungals, the orotomides. We describe the mechanism of action of olorofim, the spectrum of activity in vitro and in vivo, pharmacokinetics, pharmacodynamics, drug-drug interactions, resistance, and clinical outcomes. This comprehensive review is of utmost importance as this drugs is currently investigated in a phase III clinical trial. It has a substantial impact for the treatment of difficult to treat invasive fungal infections, such as *Lomentospora prolificans*, cryptic species of *Aspergillus*, *Scedosporium* and *Coccidioides* and for the treatment of resistant pathogens to current available antifungal drugs. Therefore this scoping review gives a clear overview on this new drug as guidance for clinicians.

We have adhered to the submission guidelines outlined by BMC Infectious Diseases and have included all necessary documents, such as the manuscript, figures and tables. Additionally, I declare that all authors have approved the manuscript for submission and that there are no issues relating to the journal policies. Hereby I confirm that the manuscript has not been published or submitted for publication elsewhere. Potential competing interests are described below in the designated section.

We are looking forward to your evaluation of your manuscript,

Yours sincerely

Dr. Yuri Vanbiervliet, MD

Prof. Dr. Johan Maertens, MD PhD

Peer reviewers

We do not have any suggestions.

Excluding peer reviewers

We would not like to exclude any peer reviewer.

Funding

This review received no external funding.

Conflict of interest

YV: has received travel support from Pfizer and Neovii.

TV: no conflicts of interest.

RA: has received a research grant and travel support from Gilead Sciences and Pfizer.

IS: has received research grants and speaker's fees from Pfizer and Gilead.

KL: received consultancy fees from MRM Health, MSD, and Mundipharma, speaker fees from Pfizer and Gilead and a service fee fromThermo Fisher Scientific and TECOmedical.

JM: has received research support from Pfizer and Gilead, and has served as a consultant or speaker for Gilead, Pfizer, MSD, Cidara, Mundipharma, F2G, Amplyx, Basilea, Shionogi, Scynexis and Takeda.
